# Supplementary material for: A single-cell RNA-seq analysis unravels the heterogeneity of primary cultured human corneal endothelial cells
Source: Sci Rep. 2023 Jun 8;13:9361. doi: 10.1038/s41598-023-36567-6 (PMC10249941; doi:10.1038/s41598-023-36567-6)
Supplement: Supplementary file 1 — Supplementary Figures. [file 41598_2023_36567_MOESM1_ESM.docx]

**SUPPLEMENTAL INFORMATION LEGENDS**

**Figure S1.** Phase contrast images of each donor time point used for scRNAseq, related to Figure 1. Phase contrast imaging shows desired endothelial morphology in all of the sequenced samples. Scale bar represents 100 μm.

**Figure S2.** UMAP projection of cells per time point, related to Figure 2. Cell distribution UMAP per each time point further confirms that cluster is composed of CEC at early culture time points. Each donor is represented by color confirming the donor distribution across cell clusters. P0D2: day 2 in proliferation media. P0D5: day 5 in proliferation media. P0D14: passage 0 maintenance media. P1D30: passage 1 maintenance media. P2D46: passage 2 maintenance media.

**Figure S3.** scRNAseq revealed absence of stroma and epithelial contamination, related to Figure 2. Gene expression UMAP of stromal markers *KERA*, *LUM*, and *ALDH1A1* and epithelial markers *KRT12*, *KRT14*, and *PAX6* confirmed absence of contaminant corneal side-populations in the primary culture.

**Figure S4.** Violin plots of differentially expressed genes for cluster identification, related to Figure 2. Violin plots of differentially expressed genes for cluster annotation. Corneal endothelium: *AP1A1, COL4A3, CHD2, ALCAM, SLC4A11, PITX2*. Senescence: *MT2A, CDKN2A, TAGLN*. Proliferation: *MKI67, CENPF, PTTG1*. Fibrosis and endothelial to mesenchymal transition: *ACTA2, CD44, COL6A1*.

**Figure S5.** Cluster 3 is enriched in ribosomal gene expression. (A) UMAP representation of ribosomal gene fraction shows that cluster 3 expresses a high amount of ribosomal genes compared to other clusters. (B) Violin plot of ribosomal gene fraction shows further confirms a high expression of ribosomal genes in cluster 3 compared to the other detected clusters.

**Figure S6.** Violin plots of differentially expressed genes used for cluster identification across various culture time points, as related to Figure 2. The plots demonstrate the gene expression for markers for endothelium (*ALCAM*, *COL4A3*), senescence (*CDKN2A*, *TAGLN*), proliferation (*MKI67*, *CENPF*), and fibrosis (*CD44*, *ACTA2*) over culture time points, namely passage 0 day 2 in proliferation media (P0D2), passage 0 day 5 in proliferation media (P0D5), passage 0 at confluency in maintenance media (P0D14), passage 1 at confluency in maintenance media (P1D30), and passage 2 at confluency in maintenance media (P2D46).

**Figure S7.** UMAP projection of cells per time point at confluency, related to Figure 3. Cell distribution UMAP per each time point at confluency level shows homogeneous distribution of donors across all cell clusters. P0D14: passage 0 maintenance media. P1D30: passage 1 maintenance media. P2D46: passage 2 maintenance media.

**Figure S8.** Violin plots of differentially expressed genes for cluster identification, related to Figure 3. Violin plots of differentially expressed genes for cluster annotation at the confluency time points. Corneal endothelium: *SLC4A11*, *ATP1A1*, *ALCAM*, *CDH2*. Corneal endothelium extracellular matrix: *COL4A8*, *COL4A3*, *COL4A1*, *COL4A2*, *COL5A2*. Senescence: *CDKN2A*, *TAGLN*, *MT2A*, *CDKN1A*, *LGALS1*. Cell secretion: *GOLGA8A*. Proliferation: *MKI67*, *CENPF*, *PTTG1*. Fibrosis and endothelial to mesenchymal transition: *COL6A3*, *CD44*, *FBLN5*, *COL6A1*, *ACTA2*

**Figure S9.** Violin plots of differentially expressed genes used for cluster identification over culture time points, as related to Figure 3. The plots exhibit the gene expression for markers for endothelium (*SLC4A11*, *ALCAM*, *ATP1A1*), proliferation (*MKI67*), senescence (*CDKN2A*, *TAGLN*), fibrosis (*CD44*, *ACTA2*), and extracellular matrix production (*COL4A2*) over culture time points, namely passage 0 at confluency in maintenance media (P0D14), passage 1 at confluency in maintenance media (P1D30), and passage 2 at confluency in maintenance media (P2D46).

**Figure S10.** Gene expression UMAP of *ALCAM* (CD166), *CD44* and *ACTA2*, related to Figure 3. Gene expression UMAP shows heterogeneous expression of *ALCAM*, *CD44* and *ACTA2* across cell cluster C3.

**Figure S11.** Identification of putative doublets. Putative doublets were identified with scDblFinder. The identified putative doublets were dispersed across all cell clusters and did not bias scRNAseq clustering therefore were not removed.

**SUPPLEMENTAL INFORMATION**


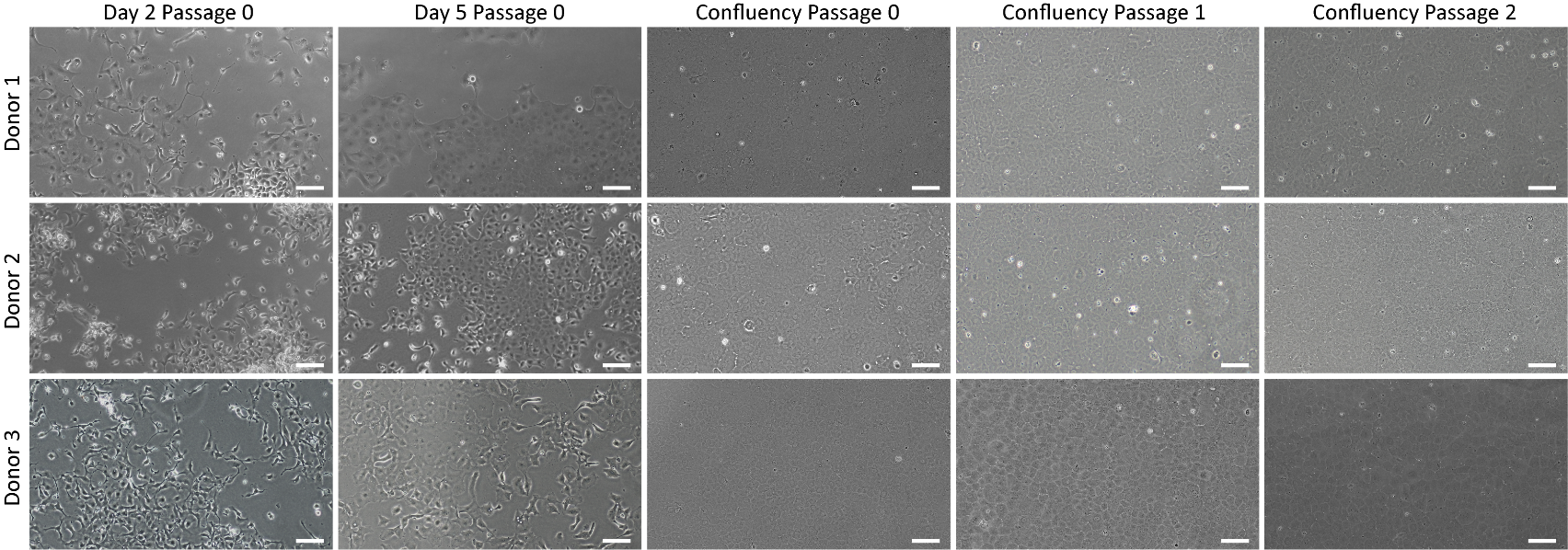
**FIGURE S1**

**FIGURE S2**

**
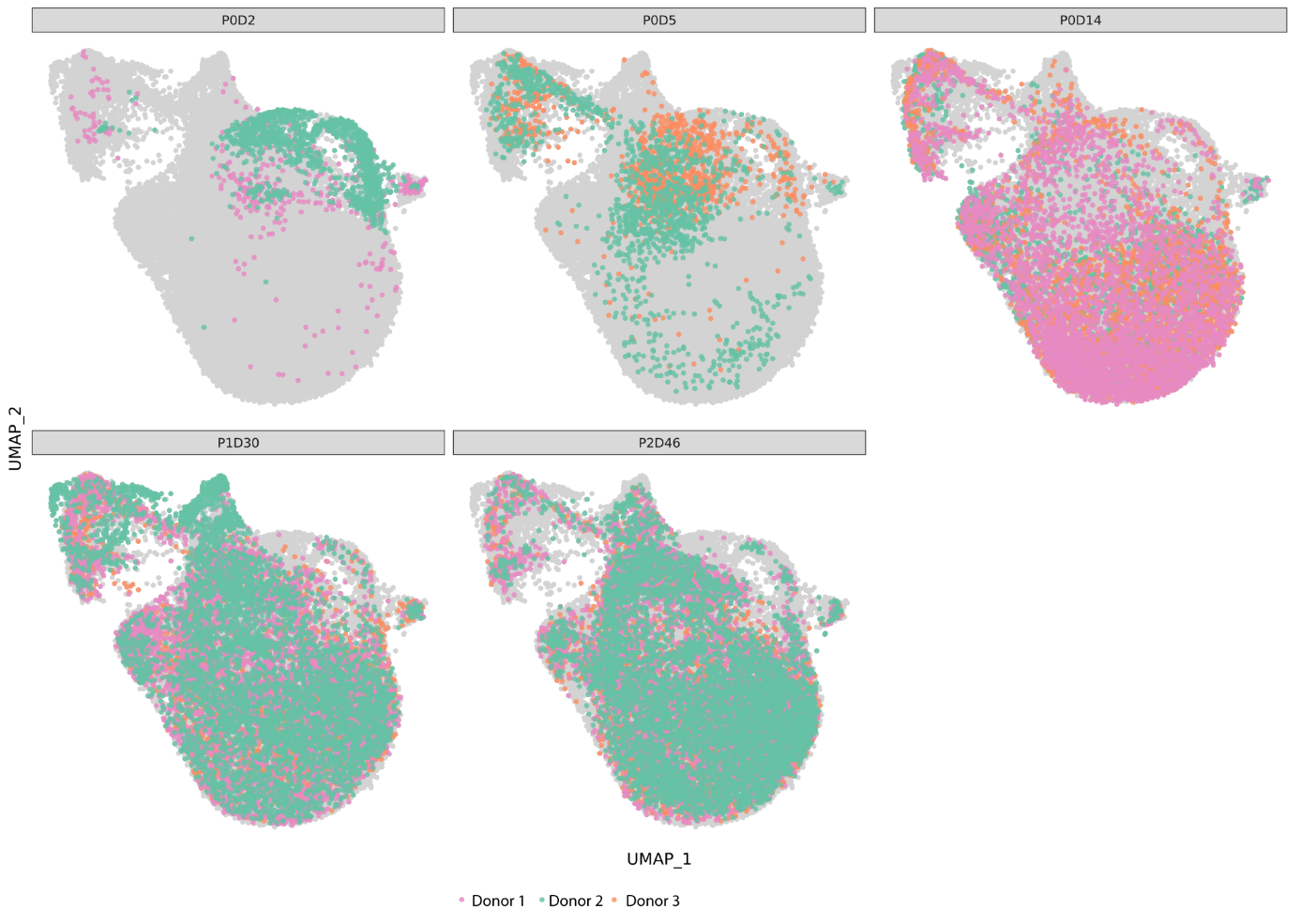
**

**FIGURE S3**

**
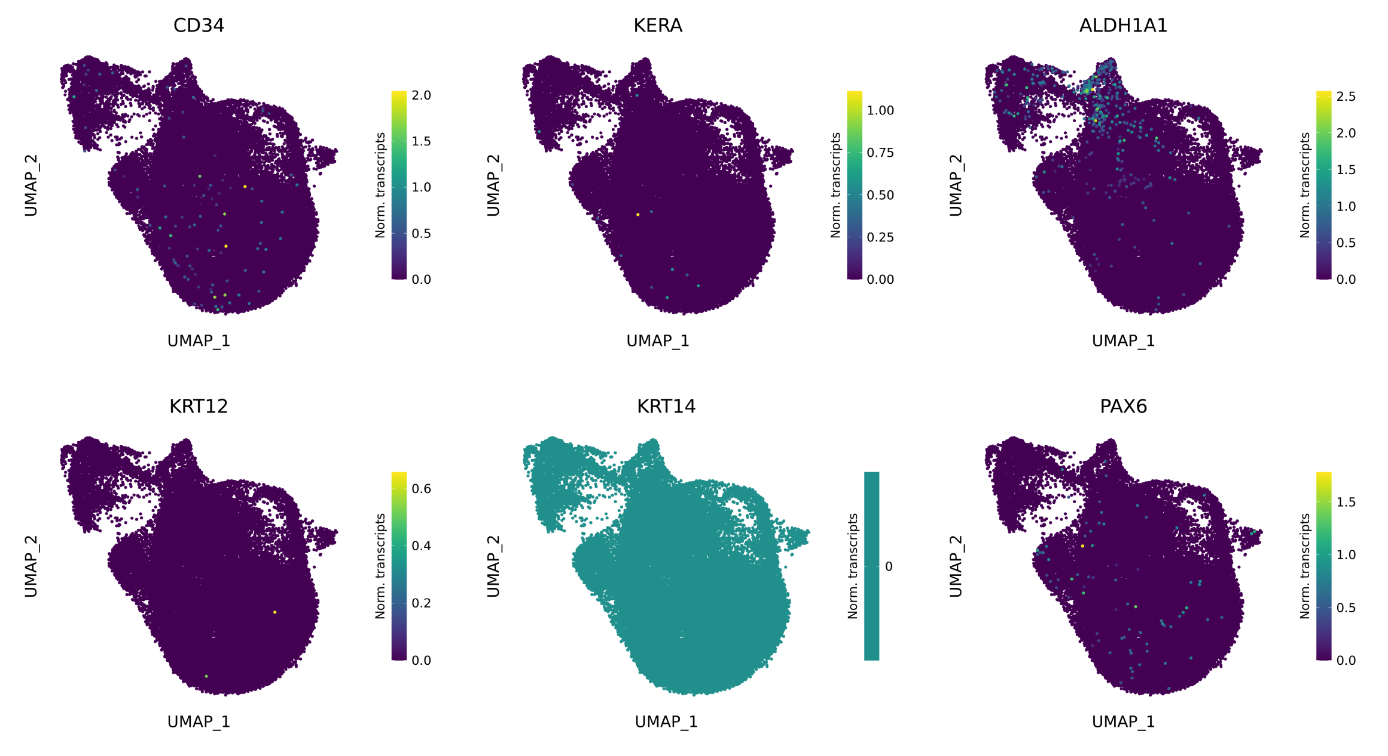
**

**FIGURE S4
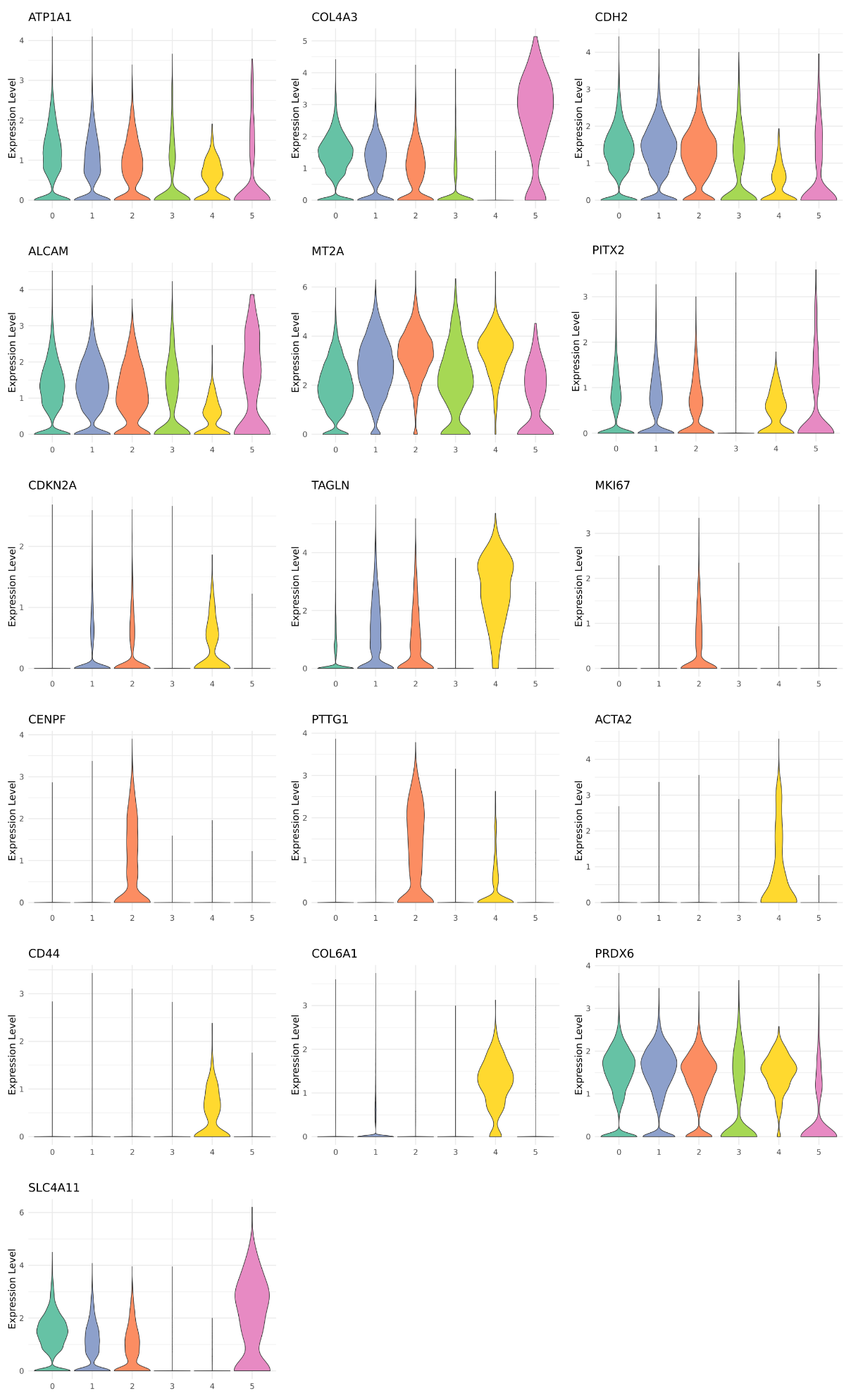
**

**FIGURE S5**

**
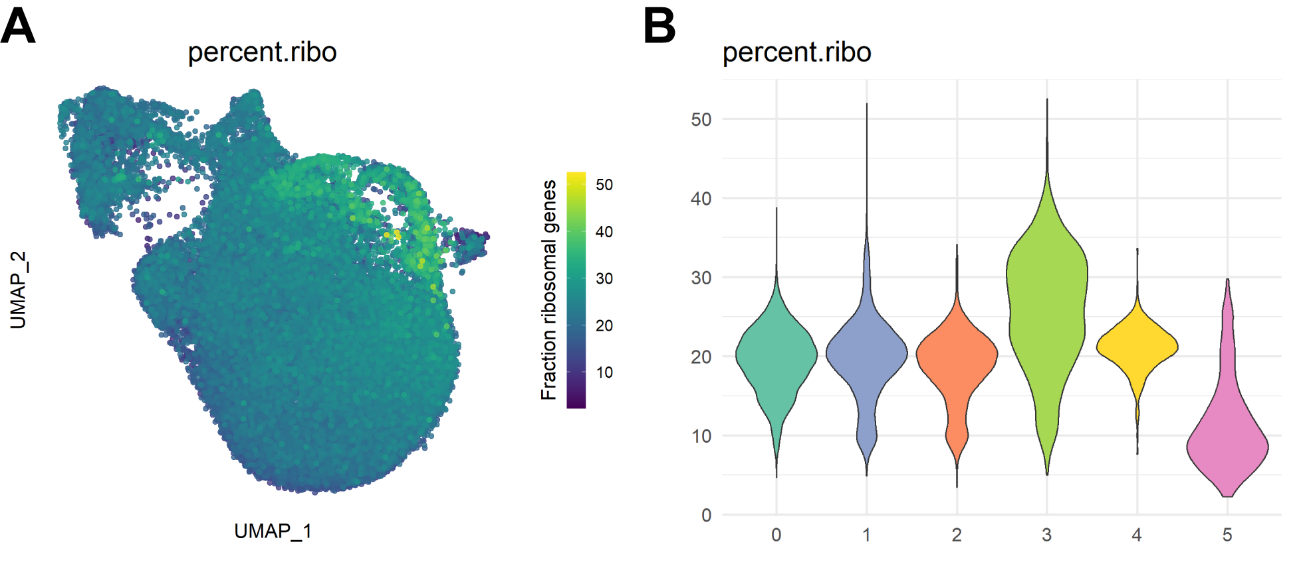
**

**FIGURE S6**

**
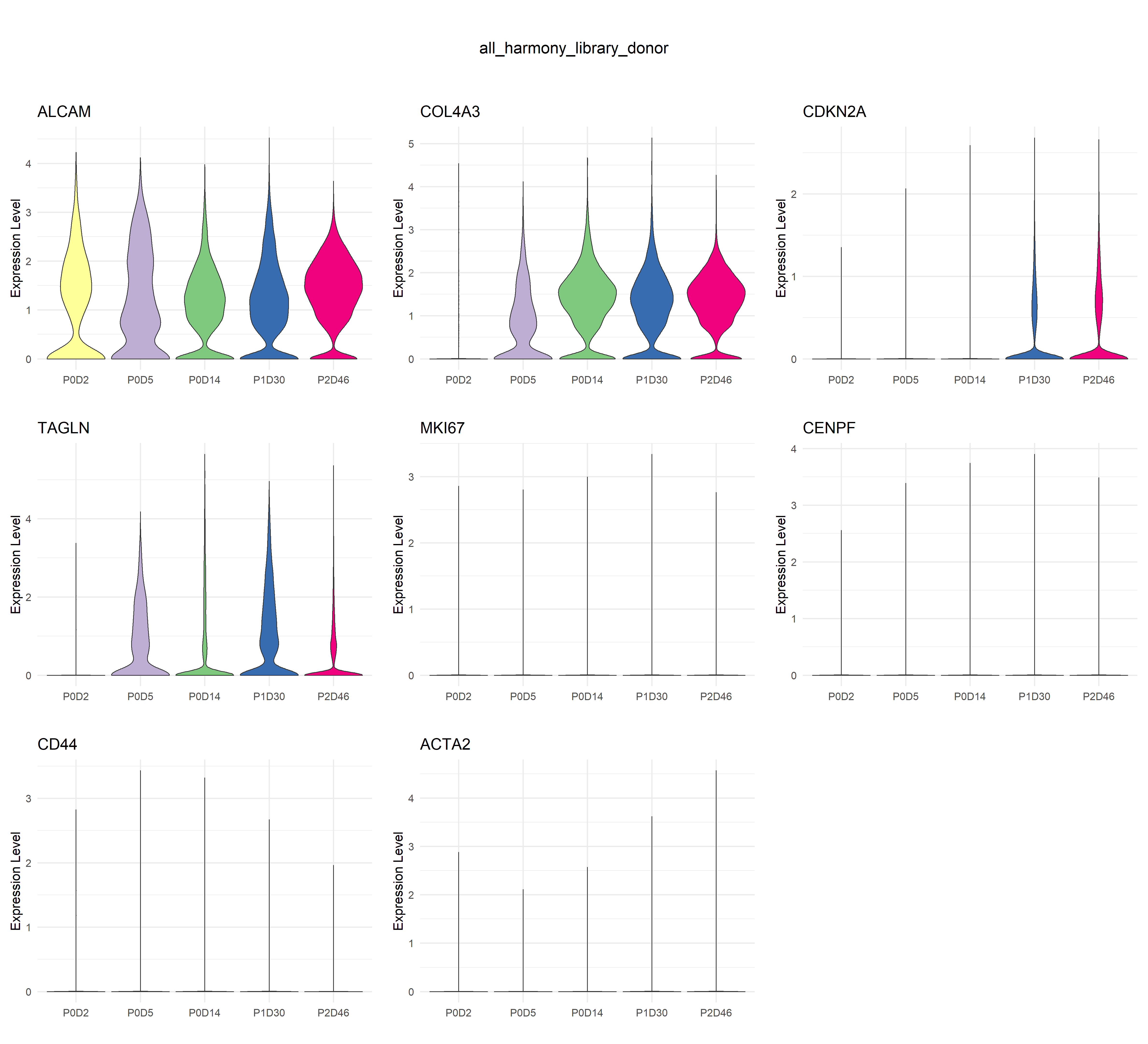
**

**FIGURE S7**

**
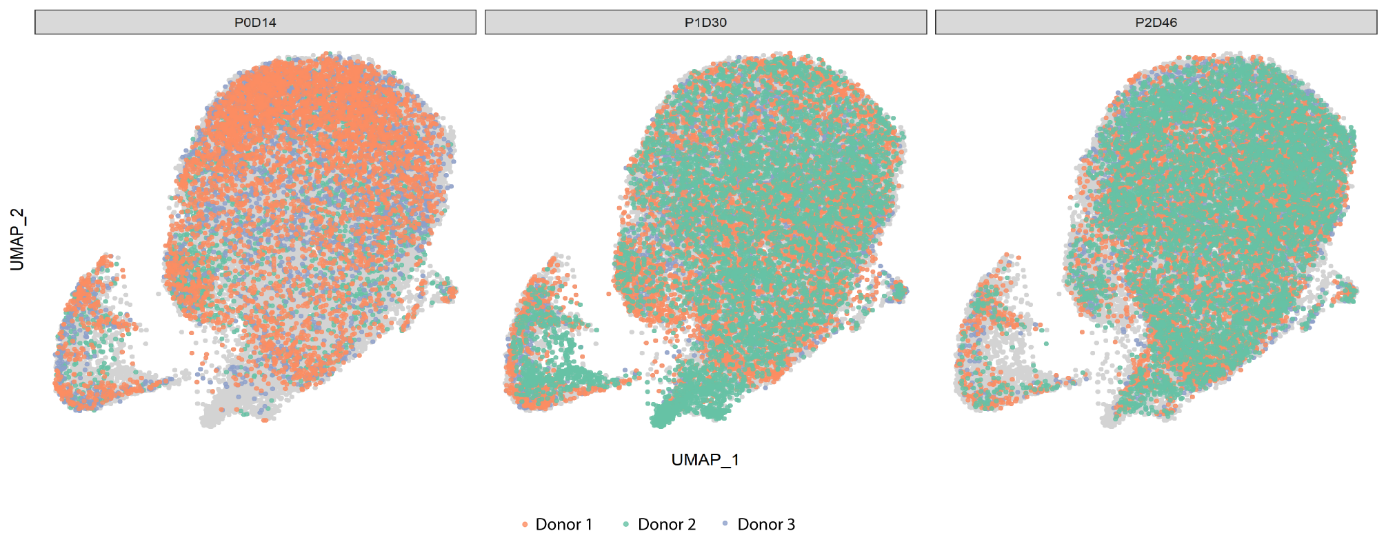
**

**FIGURE S8**

**
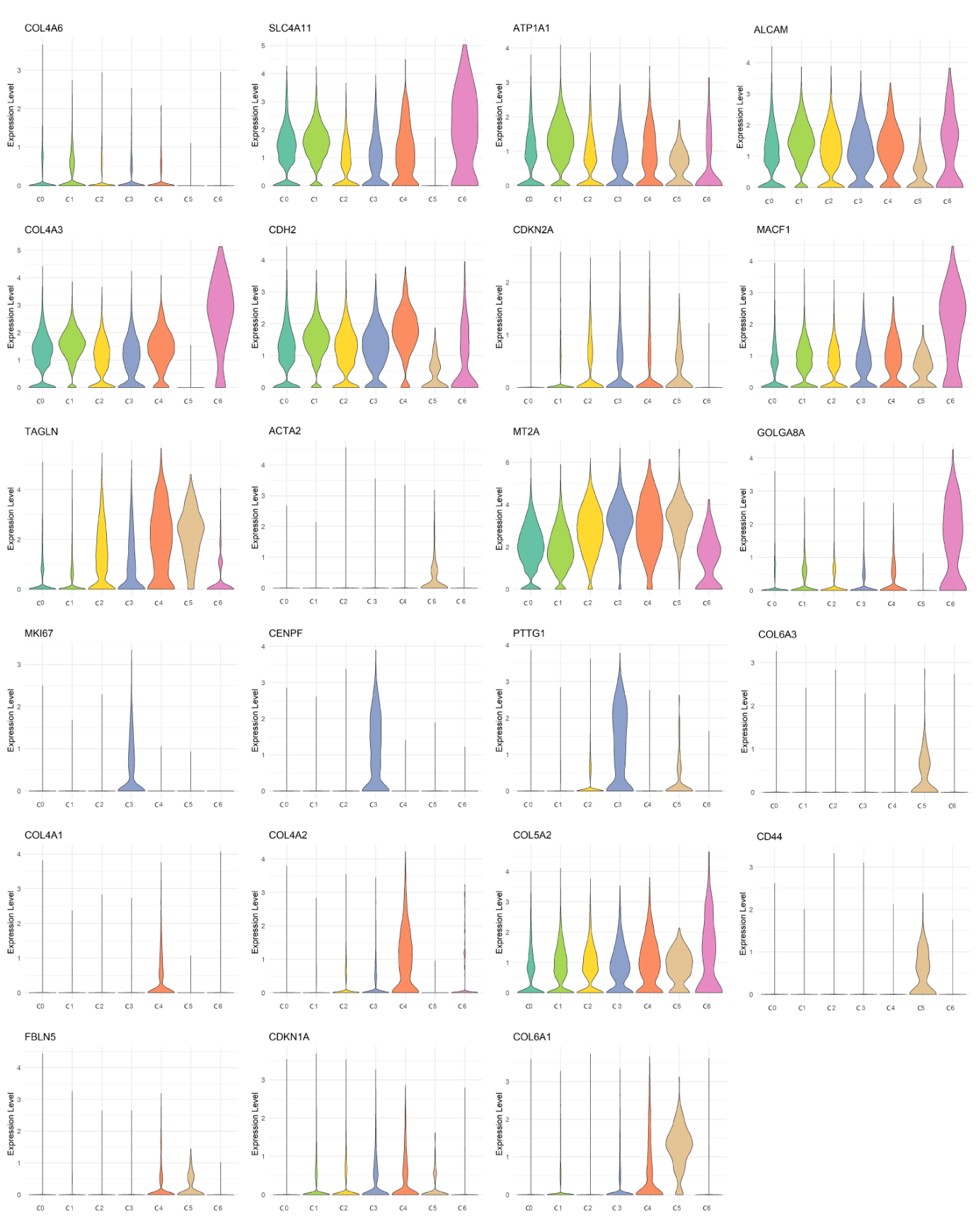
**

**FIGURE S9**

**
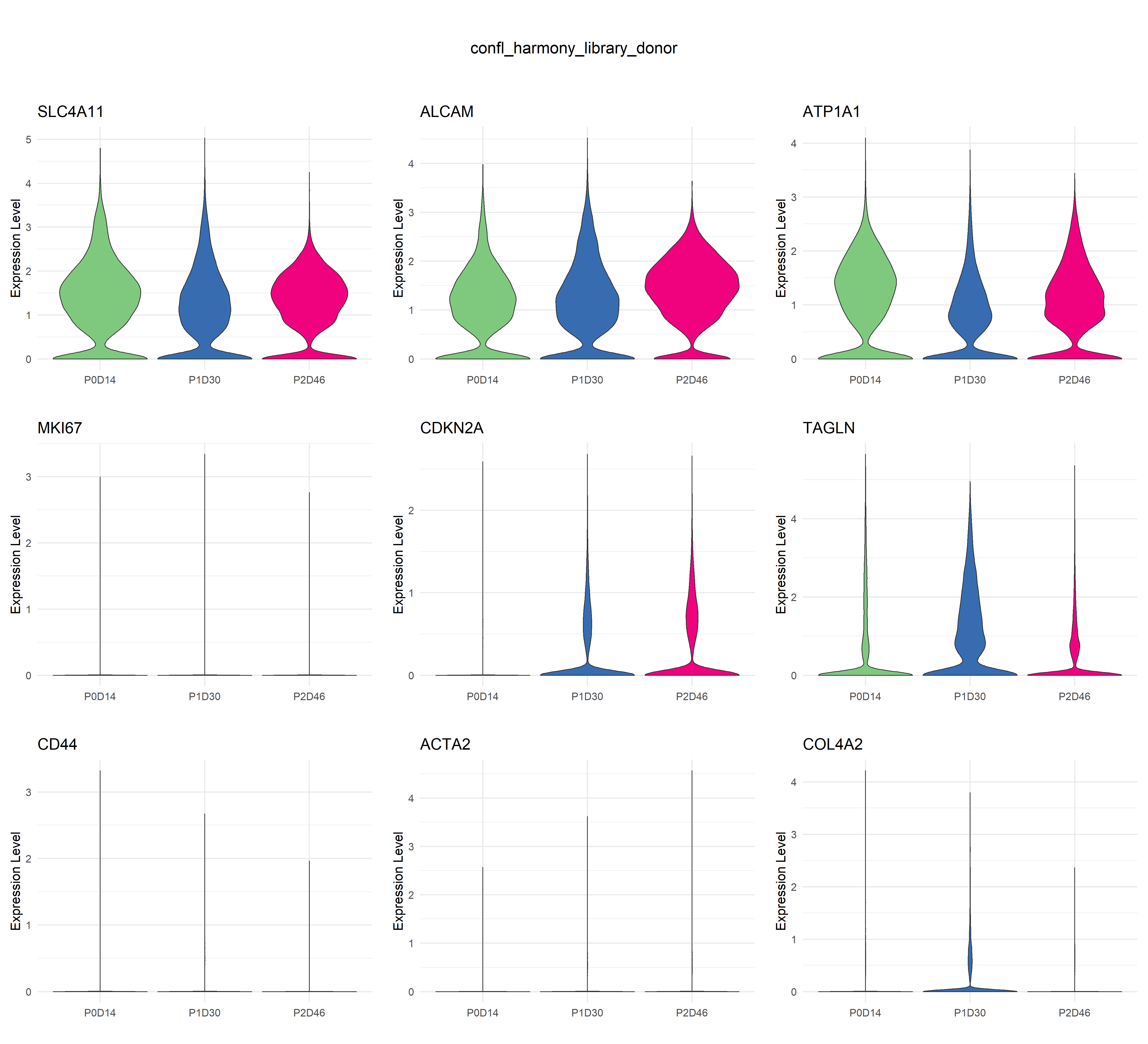
**

**FIGURE S10**

**
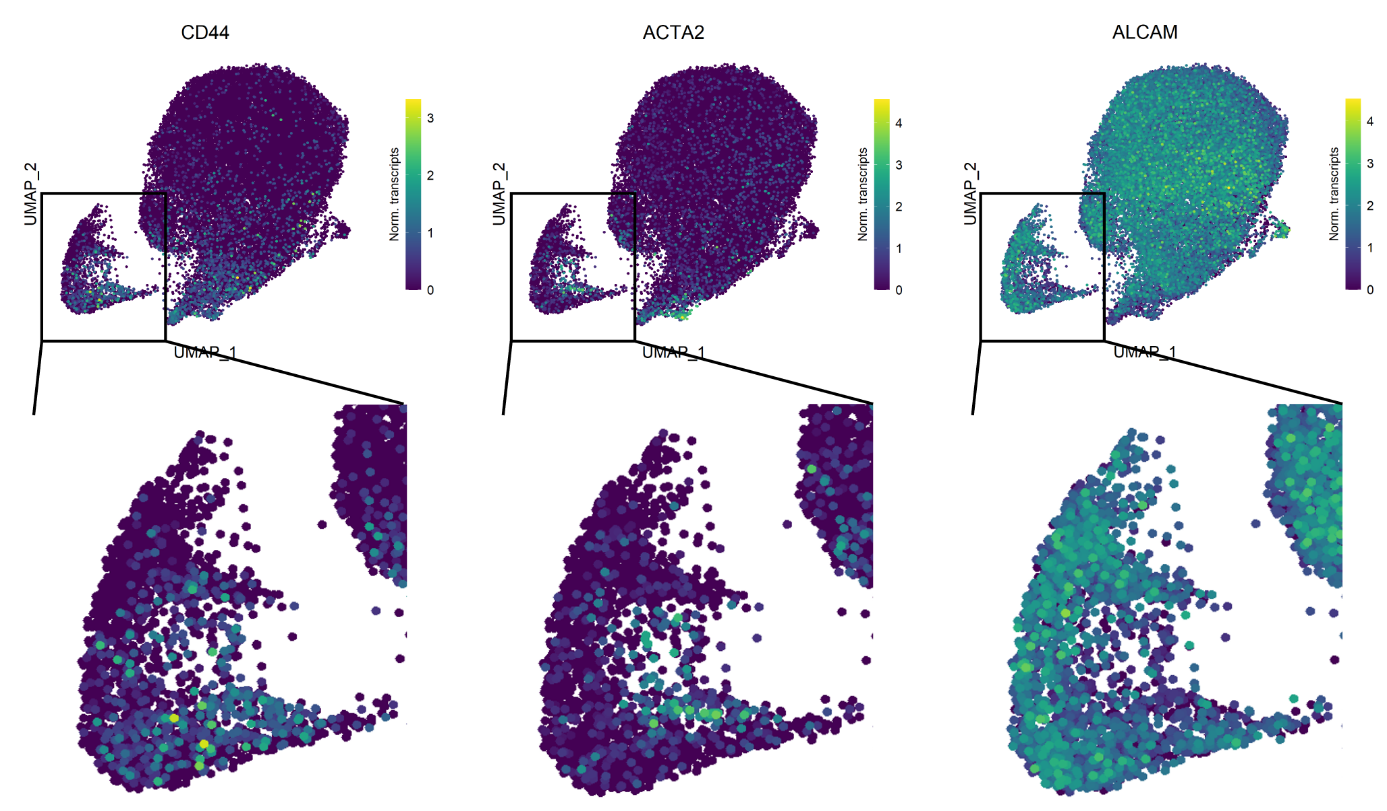
**

**FIGURE S11**

**
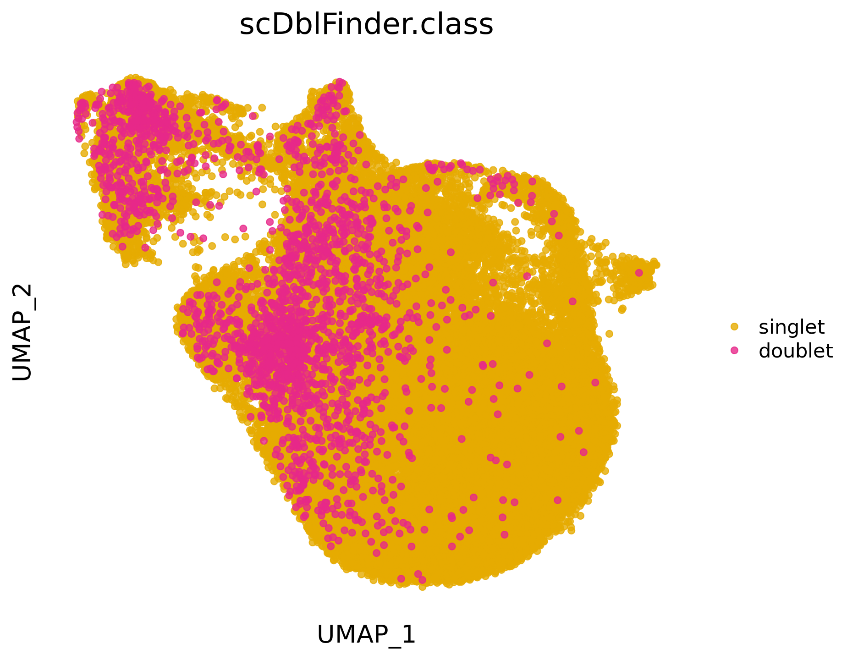
**
